# Development and validation of a preoperative nomogram for predicting patients with impacted ureteral stone: a retrospective analysis

**DOI:** 10.1186/s12894-021-00904-6

**Published:** 2021-10-08

**Authors:** Chenglu Wang, Lu Jin, Xinyang Zhao, Boxin Xue, Min Zheng

**Affiliations:** 1 Reproductive Medicine Center, Department of Reproductive Endocrinology, Affiliated People’s Hospital, Zhejiang Provincial People’s Hospital, Hangzhou Medical College, No.158 Shangtang Road, Hangzhou, 310014 Zhejiang People’s Republic of China; 2 Department of Urology, Second Affiliated Hospital of Soochow University, Suzhou, Jiangsu People’s Republic of China

**Keywords:** Impacted stone, Nomograms, Ureteroscopy, Urolithiasis, Computed tomography (CT)

## Abstract

**Background:**

To develop and validate a practical nomogram for predicting the probability of patients with impacted ureteral stone.

**Methods:**

Between June 2020 to March 2021, 214 single ureteral stones received ureteroscopy lithotripsy (URSL) were selected in development group. While 82 single ureteral stones received URSL between April 2021 to May 2021 were included in validation group. Independent factors for predicting impacted ureteral stone were screened by univariate and multivariate logistic regression analysis. The relationship between preoperative factors and stone impaction was modeled according to the regression coefficients. Discrimination and calibration were estimated by area under the receiver operating characteristic (AUROC) curve and calibration curve respectively. Clinical usefulness of the nomogram was evaluated by decision curve analysis.

**Results:**

Age, ipsilateral stone treatment history, hydronephrosis and maximum ureteral wall thickness (UWT_max_) at the portion of stone were identified as independent predictors for impacted stone. The AUROC curve of development and validation group were 0.915 and 0.882 respectively. Calibration curve of two groups showed strong concordance between the predicted and actual probabilities. Decision curve analysis showed that the predictive nomogram had a superior net benefit than UWT_max_ for all examined probabilities.

**Conclusions:**

We developed and validated an individualized model to predict impacted ureteral stone prior to surgery. Through this prediction model, urologists can select an optimal treatment method and decrease intraoperative and postoperative complications for patients with impacted ureteral calculus.

## Background

Urinary lithiasis has plagued more than 12% of the worldwide population during last several decades [1]. Impacted ureteral stones are defined as the stones staying in the same location for a prolonged time and causing ureteral obstruction [2]. Stone impaction is common in clinical practice and often lead to obstructive hydronephrosis, which may cause urosepsis even renal failure. It was reported that a patient with impacted stone caused urosepsis and required amputation of all 4 extremities [3]. Impacted stones are more common with intraoperative and postoperative complications because of the longer operation time and more complicated surgical procedures compared with non-impacted ones [4, 5]. Therefore, there is an urgent need for developing a tool to predict stone impaction prior to surgery. In previous research, ureteral wall thickness at the portion of impacted stone was identified as a critical predictor for SWL outcome [6]. Sarica et al. [7] indicated that the serum acute phase reactants CRP (C-reactive protein) and ESR (erythrocyte sedimentation rate) values as well as the UWT were helpful to estimate the presence of stone impaction. In recent, it was found that several preoperative factors, such as age, stone location and UWT, were strongly associated with the impacted ureteral stone [8]. Based on a review of previous studies, the present study aimed to evaluate the ability of preoperative factors for screening and identifying the stone impaction prior to surgery. In addition, established models for predicting ureteral calculi associated with urosepsis exist but the model for predicting the ureteric stone impaction is still in development [9]. Therefore, we subsequently developed and validated a preoperative model for predicting these stones.

## Methods

### Patients

After getting approval from institutional review board, we retrospectively analyzed 376 URSL for single ureteral stone removal at The Second Affiliated Hospital of Soochow University hospital between June 2020 to May 2021. Of these 376 procedures, 80 were excluded because of the lack of preoperative NCCT. Between June 2020 to March 2021, 214 single ureteral stones received URSL were selected in development group. While 82 single ureteral stones received URSL between April 2021 to May 2021 were included in validation group.

### Data collection

**Fig. 1:**
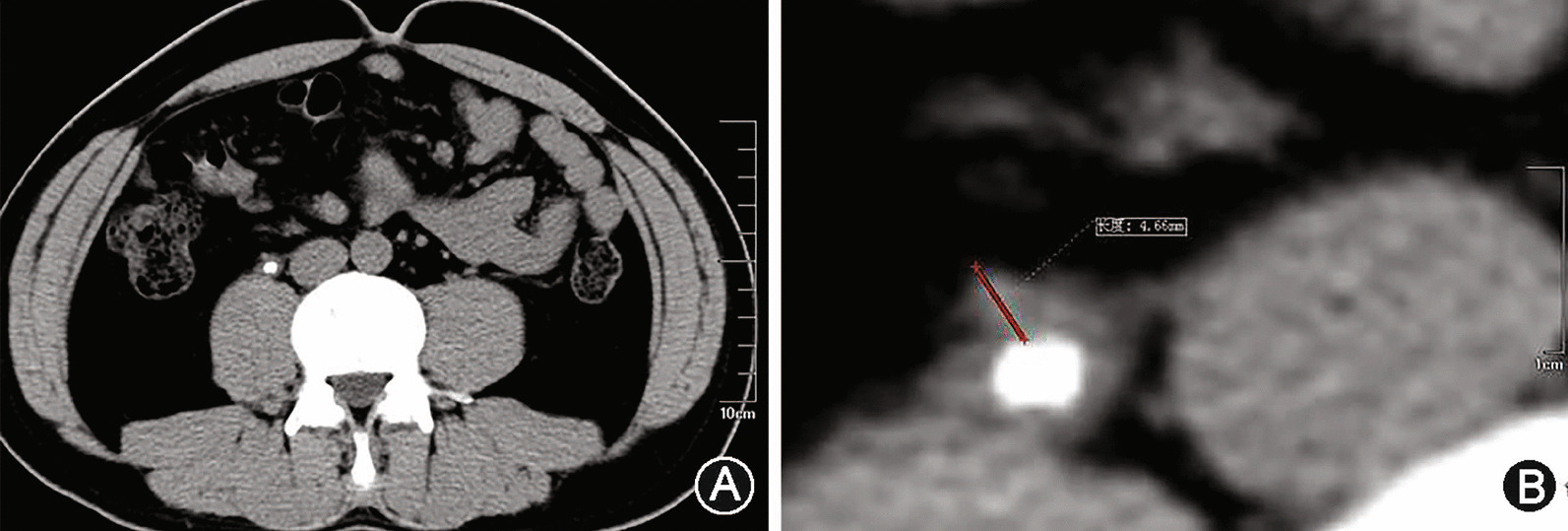
**A** The ureteral calculus is demonstrated in axial section. **B** UWT max is calculated by the imaging software. Maximal (8 ×) magnification is used to facilitate

Preoperative factors including patients baseline characteristics, laboratory values and radiological investigations were reviewed from medical records. Baseline characteristics recorded included age in years, gender, physical examination, medical history and ipsilateral stone treatment history. Stone impaction was defined intra-operatively, as failure to pass a guidewire or the smallest ureteric catheter pass the stone at 1st attempt. Patients were divided into 2 groups : Group1: impacted and Group2: Non impacted stones. Ipsilateral stone treatment history including the previous medical treatment or surgery for the same side ureteral stone. For radiologic evaluation, the preoperative NCCT was performed in all patients. Based on NCCT images, stone characteristics such as stone location, stone side, degree of hydronephrosis, maximum stone diameter, maximum cross-sectional area of the stone, stone volume, stone density (Hounsfield Unit) and UWT_max_ were reviewed by a blinded expert urologist. UWT_max_ was measured at an 8 times magnification in the axial view with an abdominal window (Fig. 1). Stone volume was estimated by scalene ellipsoid volume formula (L × W × H × π × 0.167) after measuring stone length, width and height. The degree of hydronephrosis was assigned as 0–2 values based upon follows: 0 = no or mild hydronephrosis (no calyces or renal pelvis dilation or renal pelvis dilation alone); 1 = moderate hydronephrosis (mild calyces dilation); 2 = severe hydronephrosis (severe calyces dilation).

### Surgical procedures

After spinal or general anesthesia, all URSL procedures was performed in the lithotomy position using an 8/9.5 F semi-rigid ureteroscope (Karl Storz, Tuttlingen, Germany), a holmium laser lithotripsy system (Lumenis, Santa Clara, Amercia) and a pressure-controlled irrigation pump (JingRui, Zhejiang, China). Before lithotripsy, a 0.035-inch guidewire (Terumo, Shizuoka, Japan) was employed to attempt to pass through the stone by a semi-rigid ureteroscope. If guidewire failed to pass the stone, URSL was firstly conducted for space creation between the stone and the ureteral wall. The stones were fragmented using holmium laser lithotripsy after guidewire placed in the ureter. If patients were diagnosed as proximal impacted ureteral stone, after space creating, a ureteral occlusion device was utilized for the prevention of stone migration before continuing stone fragmentation. A double J stent (size 4.7 F of 6 F) was inserted in ureter at the end of procedure. Four weeks after surgery, the stent was removed and abdominal x-ray film was taken to detect the stone-free status in the follow up evaluation. All procedures were finished by the same experienced endourologist.

### Statistical analysis

All clinical factors were compared by Mann-Whitney *U* and chi-square tests. Continuous variables are presented as mean and categorical variables are presented as percentage. The factors showing statistical significance in univariate analysis were further included in the multivariate logistic regression analysis. A variable selection method of *P* *<* 0.1 was selected for variable elimination in the model. Before variable selection, nomogram variables were coded by a blinded investigator. Based on regression coefficients of selected variables, an individualized nomogram prediction model for ureteral stone impaction was established. The predictive ability of nomogram was evaluated according to discrimination and calibration. The discrimination refers to the ability to correctly distinguish between patients with impacted stones from those without impaction. Area under the ROC curve was calculated for the assessment of model discrimination. The calibration refers to the ability of concordance between the average predicted probability and the out prevalence in patients with impacted stones. Calibration curve was used to investigate the goodness of fit for prediction model. Additionally, the clinical usefulness of the nomogram was investigated by decision curve analysis. The net benefit of decision curve analysis, combined the number of true positives and false positives, was used to evaluate the practical value of risk model.

Statistical analyses were performed using SPSS statistical software (ver 24.0, USA) and R statistical software (ver 3.5.3, USA). Two-tailed analysis with *P* *<* 0.05 was considered as statistically significant.

## Results

### Patient data

A total of 296 patients were included in the present study. The stone-free rate in development group were 97.7% (non-impacted patients) and 89.0% (impacted patients) respectively, whereas stone-free rate in validation group were 98.0% (non-impacted patients) and 87.5% (impacted patients) respectively (*P <* 0.05). All clinical characteristics were presented in Table 1. Comparison of factors in development and validation groups indicated that no significant differences were found between patients in two groups. Significant differences between the impacted and non-impacted patients in the development group were viewed for the following parameters: age (*P <* 0.001), ipsilateral stone treatment history (*P <* 0.001), stone density (*P =* 0.008), hydronephrosis (*P <* 0.001), maximum stone diameter (*P =* 0.002), maximum cross-sectional area of the stone (*P =* 0.002), stone volume (*P =* 0.002), percussion tenderness over kidney region (*P <* 0.001) and UWT_max_ (*P <* 0.001).

**Table 1:** Comparison of patients and stone characteristics according to the impacted stones and non-impacted stones

Variable	Development group (n = 214)		Validation group (n = 82)		*P* value
	Impacted (n = 82)	Non-impacted (n = 132)	Impacted (n = 32)	Non-impacted (n = 50)	
Gender (%)					0.203
Male	49 (59.8)	73 (55.3)	19 (59.3)	21 (42.0)	
Female	33 (40.2)	59 (44.7)	13 (40.6)	29 (58.0)	
Mean (SD) age, year	58.85 (12.34)	45.12 (12.78)	57.72 (12.19)	46.39 (12.92)	0.655
Hypertension (%)					0.267
Yes	29 (35.3)	40 (30.3)	8 (25.0)	13 (26.0)	
No	53 (64.6)	92 (69.7)	24 (75.0)	37 (74.0)	
Diabetes (%)					0.803
Yes	8 (9.8)	15 (11.3)	3 (9.4)	5 (10.0)	
No	74 (90.2)	117 (88.6)	29 (90.6)	45 (90.0)	
Ipsilateral stone treatment history (%)					0.885
Yes	50 (61.0)	20 (15.2)	18 (56.3)	8 (16.0)	
No	32 (39.0)	112 (84.8)	14 (43.8)	42 (84.0)	
Stone side (%)					0.543
Right	37 (45.1)	68 (51.5)	19 (59.4)	18 (36.0)	
Left	45 (54.9)	64 (48.5)	13 (40.6)	32 (64.0)	
Stone location (%)					0.707
Proximal ureter	40 (48.8)	69 (52.3)	14 (43.8)	24 (48.0)	
Middle ureter	18 (22.0)	21 (15.9)	8 (25.0)	10 (20.0)	
Distal ureter	24 (29.3)	42 (31.8)	10 (31.3)	16 (32.0)	
Hydronephrosis (%)					
NO or mild	4 (4.9)	89 (67.4)	1 (3.1)	29 (58.0)	0.252
Moderate	41 (50.0)	39 (29.5)	13 (40.6)	19 (38.0)	
Severe	37 (45.1)	4 (3.0)	18 (56.3)	2 (4.0)	
Mean (SD)					
Maximum diameter of stone, mm	8.67(2.28)	7.17(2.75)	8.52(2.54)	7.32(2.69)	0.872
Maximum cross-sectional area of the stone, mm^2^	43.83(23.65)	31.14(23.64)	41.53(25.12)	32.97(25.06)	0.619
Stone volume, mm^3^	386.20(296.60)	253.04(296.29)	373.57(292.37)	265.13(292.71)	0.335
Stone density, HU	847.66(282.39)	698.65(325.50)	858.92(281.87)	688.27(326.29)	0.329
UWT _max,_ mm	4.15(0.94)	2.58(0.76)	4.08(0.82)	2.53(0.78)	0.913
Percussion tenderness over kidney region (%)					
Positive	18 (22.0)	8 (6.1)	12 (37.5)	3 (6.0)	0.356
Weak positive	54 (65.9)	75 (56.8)	15 (46.9)	29 (58.0)	
Negative	10 (12.2)	49 (37.1)	5 (15.6)	18 (36.0)	
Stone-free rate	89.0% (73)	97.7% (129)	87.5% (28)	98.0% (49)	0.871

### Nomogram development

**Fig. 2:**
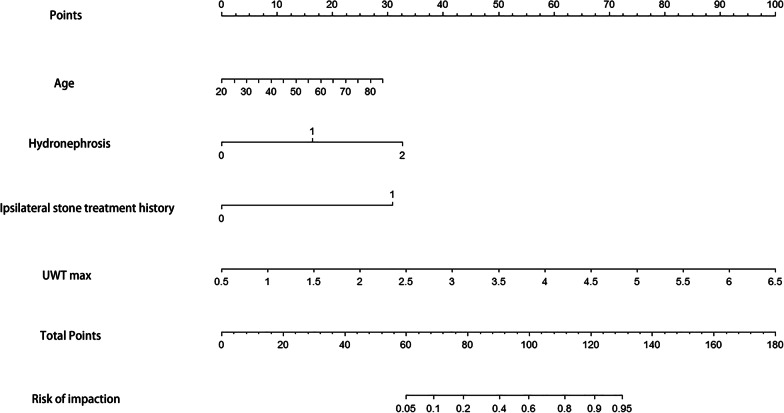
Preoperative nomogram for predicting patients with impacted stone

Statistically significant factors screened from the univariate analysis were further compared by multivariate logistic regression analysis. The four variables including age (*P =* 0.046), degree of hydronephrosis (*P =* 0.026), ipsilateral stone treatment history (*P <* 0.001) and UWT_max_ (*P =* 0.001) were identified as independent risk predictors of impacted stones (Table 2). Based on multivariate logistic regression analysis, the prediction model was established using the abovementioned four preoperative characteristics. An individualized nomogram was then developed (Fig. 2). According to the nomogram, the total score was obtained from the individual score of each prediction indicator, and the predicted risk corresponding to the sum of points is viewed as the probability of stone impaction.

**Table 2:** Univariate and multivariate logistic regression analysis of impacted prediction

Variable	Univariate analysis		Multivariate analysis	
	OR (95 %CI)	*P* value	OR (95 %CI)	*P* value
Gender	1.20 (0.69–2.10)	0.253	NA	
Age	1.12 (1.07–1.16)	< 0.001	1.06 (1.00–1.13)	0.046
Hypertension	1.26 (0.70–2.26)	0.441	NA	
Diabetes	0.84 (0.34–2.09)	0.712	NA	
Ipsilateral stone treatment history	8.75 (4.57–16.77)	< 0.001	5.20 (2.17–12.79)	< 0.001
Stone side	0.77 (0.45–1.35)	0.363	NA	
Stone location	1.48 (0.71–3.10)	0.300	NA	
Hydronephrosis	15.31 (7.41–31.62)	< 0.001	6.14 (1.22–11.29)	0.026
Maximum diameter of stone	1.24 (1.06–1.46)	0.002	NA	
Maximum cross-sectional area of the stone	1.02 (1.01–1.04)	0.002	NA	
Stone volume	1.00 (1.00–1.00)	0.002	NA	
Stone density	1.00 (1.00–1.00)	0.008	NA	
UWT max	10.17 (4.20–24.60)	< 0.001	6.23 (2.02–19.22)	0.001
Percussion tenderness over kidney region	0.91 (0.03–0.27)	< 0.001	NA	

### Nomogram validation

**Fig. 3:**
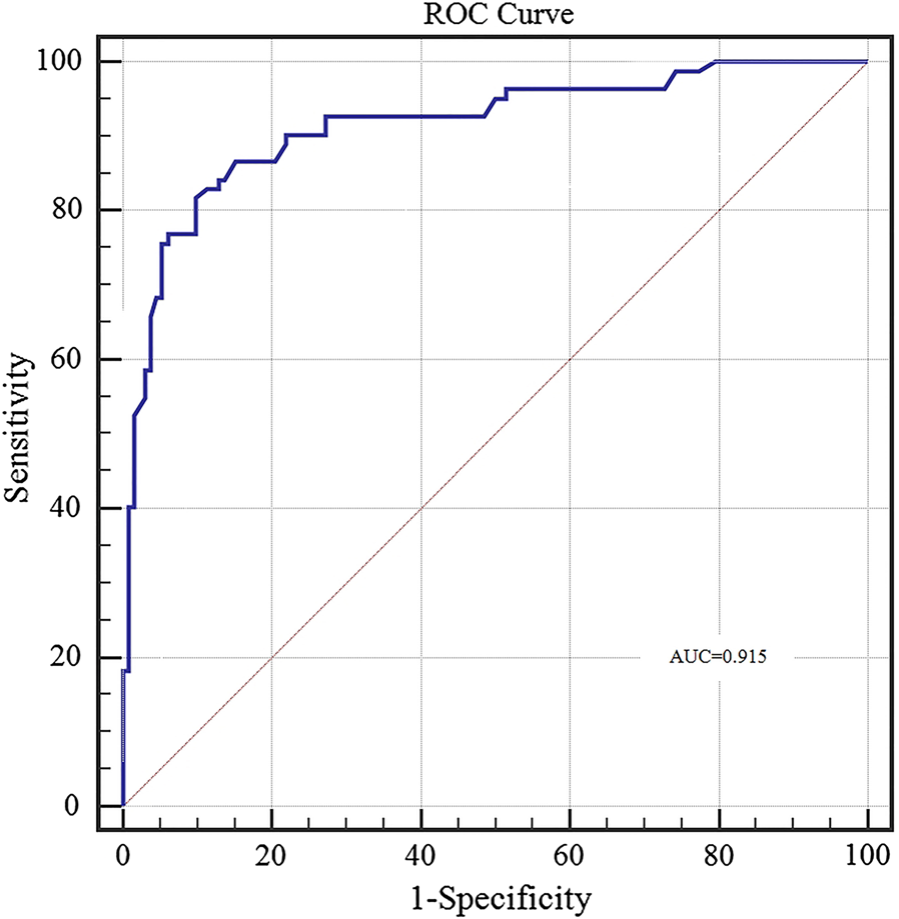
The ROC AUCs of the development group

**Fig. 4:**
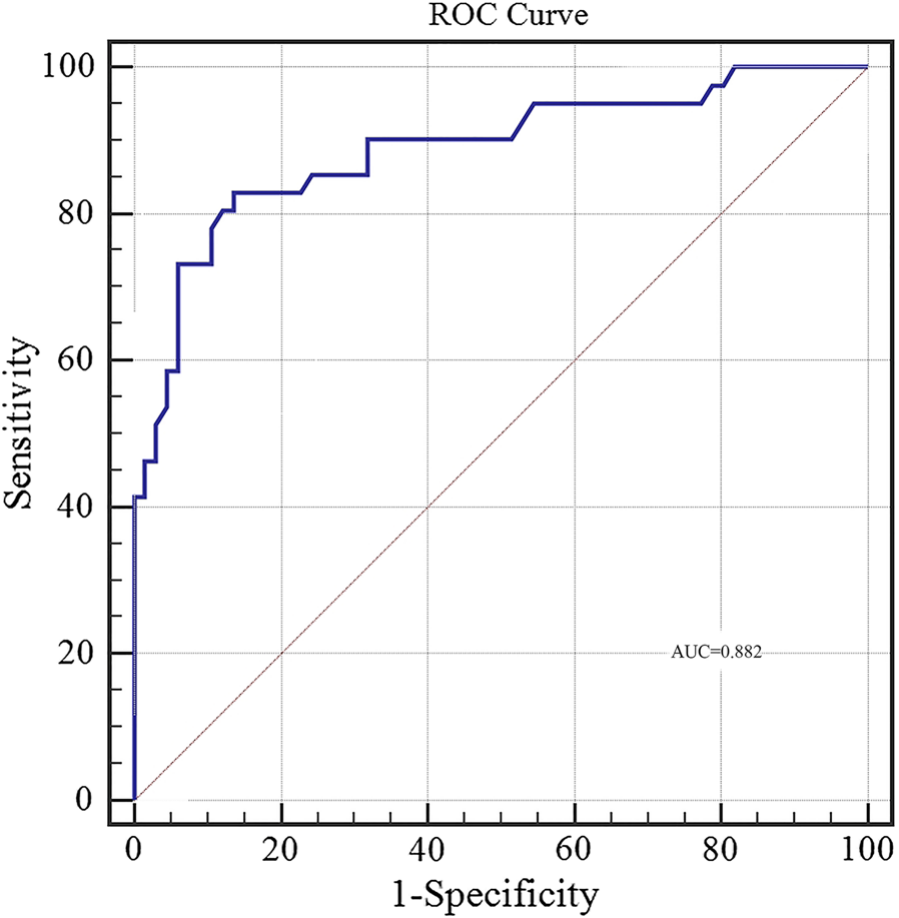
The ROC AUCs of the validation group

**Fig. 5:**
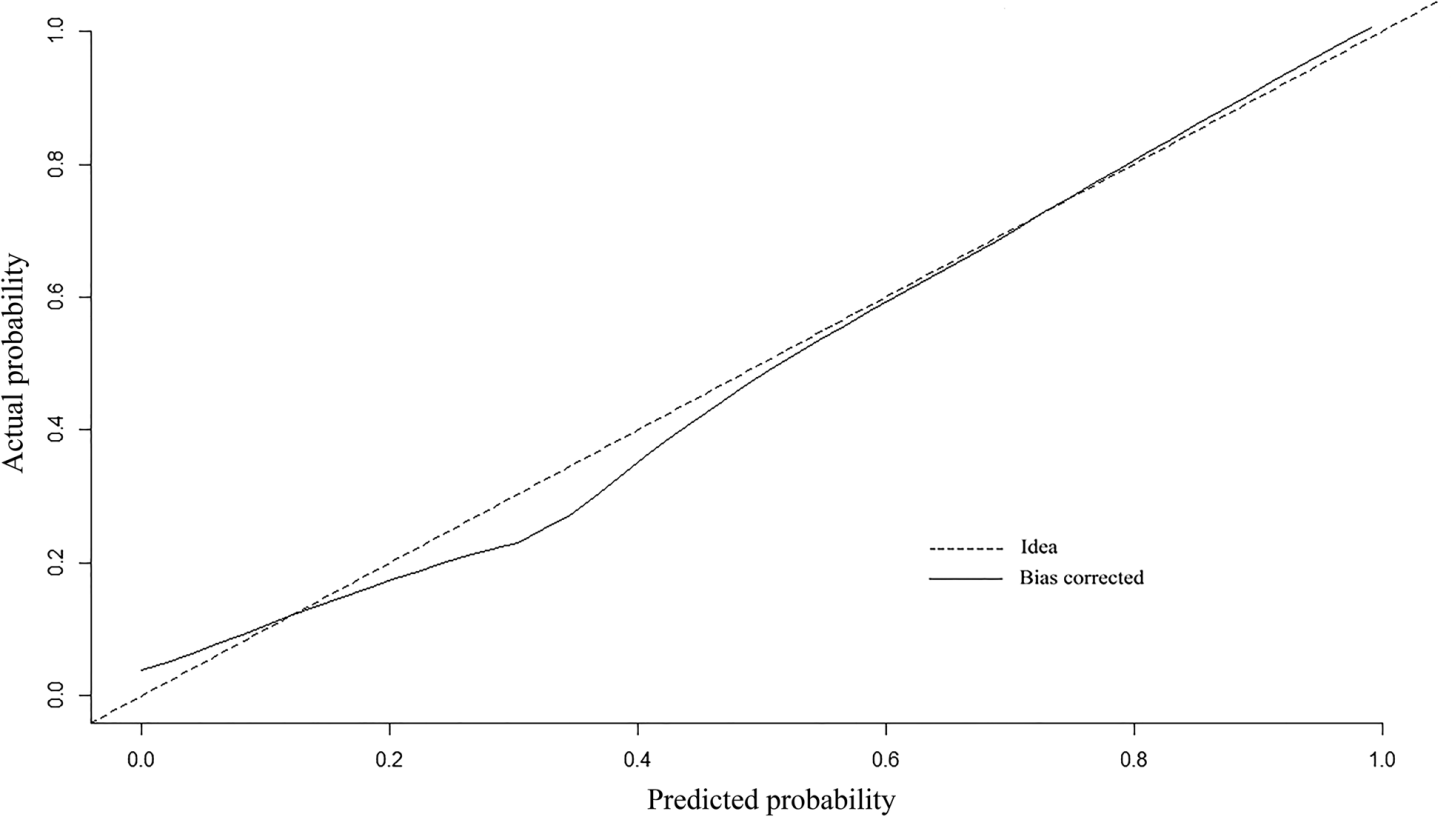
The calibration curve of the actual vs. predicted probability of the preoperative nomogram for predicting impacted ureteral stone in the development group

**Fig. 6:**
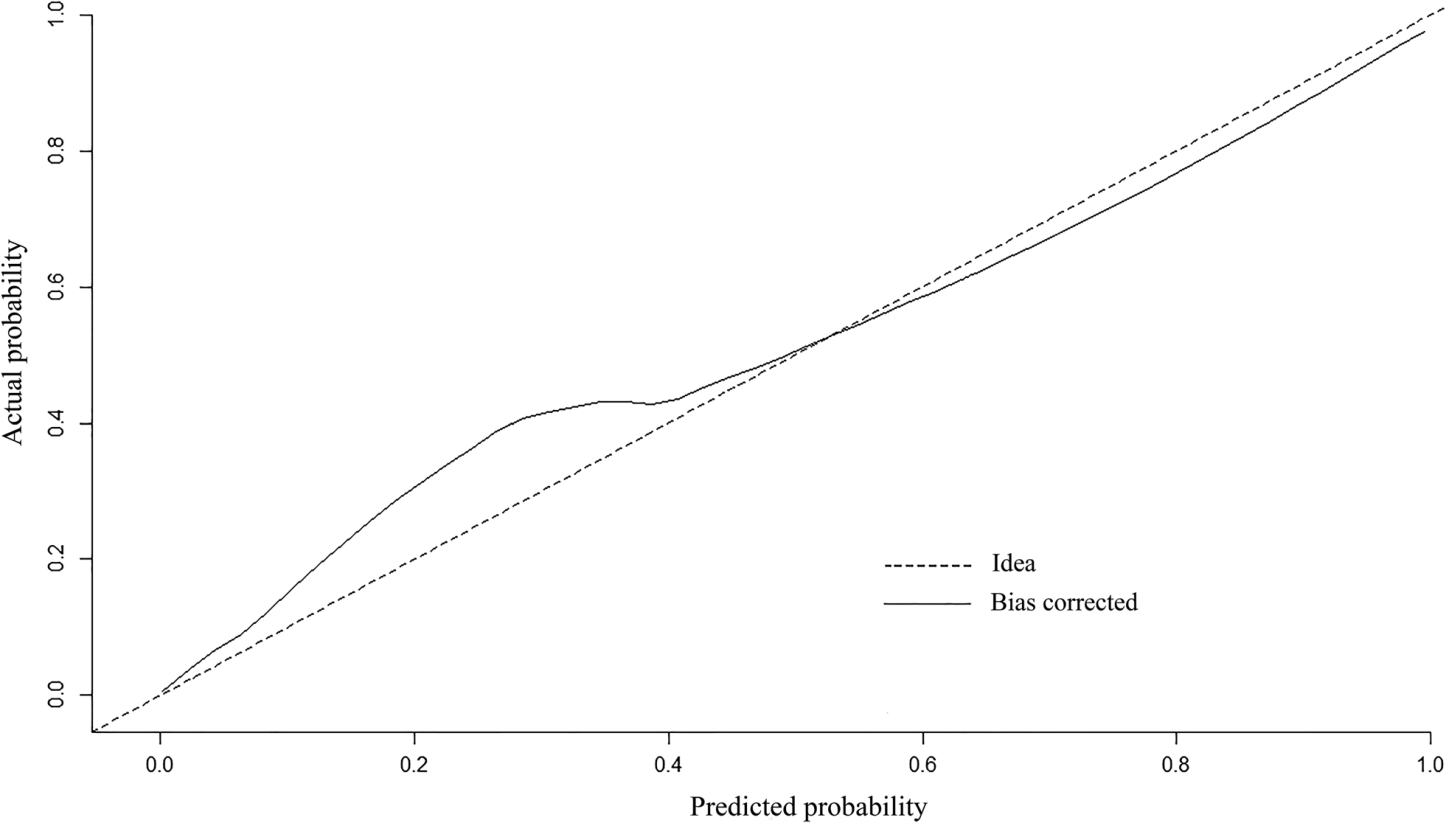
The calibration curve of the actual vs. predicted probability of the preoperative nomogram for predicting impacted ureteral stone in the validation group

**Fig. 7:**
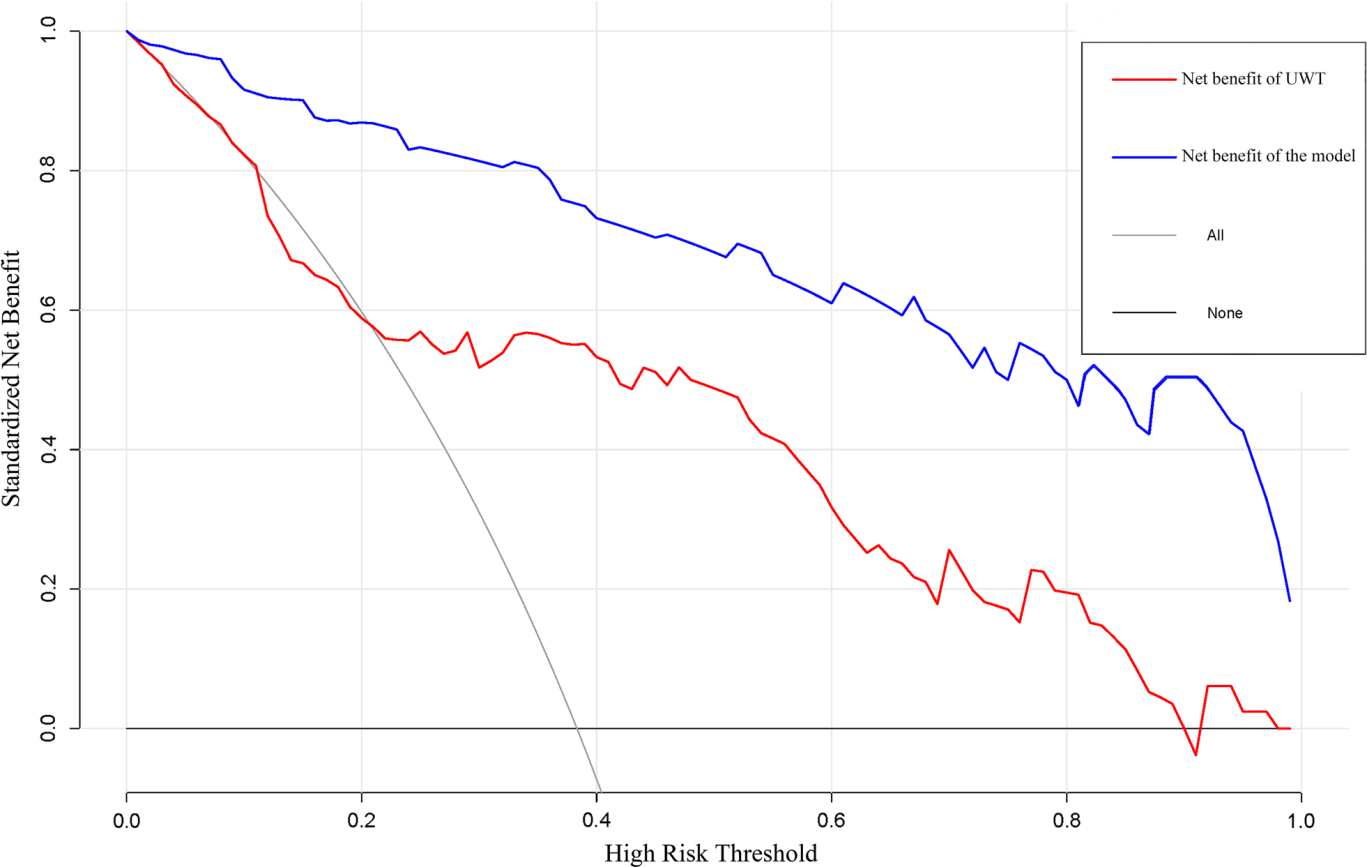
The DCA of the nomogram

The predictive ability of the model was evaluated according to discrimination and calibration. ROC curves along with AUC values were used to access the accuracy of the model in development and validation group. The estimated AUC values for impaction risk of these two groups were 0.915 (Fig. 3) and 0.882 (Fig. 4) respectively, demonstrating excellent discrimination for the predictive model. Calibration curve of the present model in both groups showed strong concordance between the predicted and actual probabilities (Figs. 5 and 6). Moreover, decision curve analysis reviewed that the practical nomogram had a superior net benefit than that risk predicting with UWT_max_ for all of the examined probabilities (Fig. 7).

## Discussion

Ureteral calculous disease is highly prevalent and affects both males and females in all age groups. Impacted ureteral stone is defined as the calculi staying at the same place in ureter for at least 2 months, causing inflammatory changes in the ureteral mucosa and resulting in epithelial hypertrophy as well as extensive ureteral edema [2]. For a prolonged period of obstruction, impacted stone may cause pyonephrosis, urosepsis, and even severe renal failure. Such stone is not uncommon in clinical practice. Thus, early diagnosis and treatment of impacted ureteral stone to avoid serious complications and consequences is essential for urologists. However, the development of new diagnostic methods had seen little progress during the last three decades. Previously, stone impaction has been defined as the calculi that has not progressed proximally or distally for more than 2 months. Nevertheless, it is challenge for urologists to determine precisely the duration of calculi in the ureter. In addition, some researchers have defined an impacted stone as the antegrade or retrograde contrast cannot readily pass through it [10]. Nonetheless, using this method may precludes the opportunity for the selection of suitable surgical procedures because urologists cannot discover the impaction until the time of surgery.

Reviewing several studies published in the last few decades, UWT has aroused much attention since it was initially reported. After analyzing the clinical factors of 80 patients with single proximal ureteral stone, Kemal and colleagues found that the value of CRP, ESR and UWT are associated with the presence of ureteric stone impaction [7]. They also noted that UWT can serve as a predictor for the final SWL outcome in patients with impacted proximal ureteral stones [6]. The recently-introduced study indicated that applying a cut-off of 3.49 mm, high UWT is closely related with a higher risk of stone impaction as well as more adverse outcomes of URSL [8].

Consistent with previous study, UWT_max_ was found as one of the independent pretreatment predictors for stone impaction in the present study. Apart from UWT_max_, multivariate logistic regression analysis suggested that age in years, ipsilateral stone treatment history and degree of hydronephrosis were also as the preoperative independent predictors. Previous research found that ureteral hypertrophy, interstitial fibrosis as well as secretion of fibrinous exudate were basic pathologic changes of impacted ureteral stone [11, 12]. Endoscopic features including inflammatory ureteral polyps and ureteral stricture have been described by Mugiya et al. [12] Because of large stone burden and long period of obstruction, server inflammatory and immune response at the ureteral mucosa were recognized as the pathological causes of impaction [13]. The above-mentioned chronic and repeat episodes can further cause fibroepithelial ureteral polyps and ureteral stricture. Bolton et al. [13] retrospectively evaluated the records of 140 patients who were diagnosed as fibroepithelial ureteral polyps and discovered concomitant urolithiasis in 10 cases. Roberts et al. [2] found that 24% patients was associated with ureteral stricture during 5 to 17 months of stone impaction.

Except urinary calculi, there were several important risk factors including urinary crystals, stents, infection and residual fragments were identified as reasons for inflammatory response of ureteral tissues. Among four preoperative independent predictors, ipsilateral stone treatment history especially URSL was one of the most important factors which has strong correlation with the ureteral polyps and stricture. During URSL, ureteroscope was introduced into ureter by a guide wire and varying degrees of injury can be obtained by this invasive manipulation. Other operative risk events such as residual fragments after surgery, thermal tissue injury by the holmium and double J stent placement after stone extraction can happen during URSL. In view of the importance of stone treatment history, therefore, we hypothesized that the stone impaction can be avoided by minimizing intraoperative injury and reducing the residue rate of stone.

Extracorporeal shock wave lithotripsy (SWL), ureteroscopy lithotripsy (URSL), and percutaneous nephrolithotomy (PCNL) are the available noninvasive management strategies for ureteral stones [14]. Nonetheless, treatment of impacted ureteral stone remained a controversial issue for the urologists and the optimal treatment method for such stone still needs to be established. Compared with non-impacted stone, disintegration of impacted stone is more difficult because of the tissue changes at the surrounding ureteral wall and the lack of natural expansion space. Therefore, impacted ureteral calculi are considered as a high-risk factor for treatment failure, stone residue as well as the occurrence of intraoperative and postoperative complications [5]. Published studies so far have clearly demonstrated that SWL is a first line choice with acceptable and effective results for proximal ureteral stones [15, 16]. However, stone-free rates are greatly decreased as stone impaction with reported success rate of 45–60% [17–19]. With the predictive model for identifying proximal impacted ureteral stone before management, urologist could choose the antegrade approach strategies for these stones. Moufid et al. [20] reported that antegrade approach had significantly higher stone-free rate (95.5% v 66.7%) and lower failure rate (0% *v* 33%) for impacted upper ureteral stone > 15 mm. Long et al. [21] retrospectively reviewed 163 patients after treatment of mini-PCNL (MPCNL) and found 95.7% stone-free rate without any complications such as ureteral perforations or postoperative strictures in 6 months follow-up. In addition, a comparative study carried out by Wang et al. [22] showed that outcomes of MPCNL and retroperitoneal ureterolithotomy (RPUL) were more favorable than URSL in patients with impacted upper ureteral stone. Consequently, instead of URSL, MPCNL and RPUL are more recommended as suitable procedures if the patients’ impaction risk predicted from the nomogram is high. Nephrostomy tube placement before URSL allows for decompression of the obstructed renal collecting system with minimal complications. This treatment procedure also could be considered as an option in case of impacted stones. Additionally, in a recently published study, tip-flexible semirigid ureterorenoscope was introduced as a novel management strategy for safely and effectively treating impacted stones with a low rate of complications [23]. Moreover, a report in recent research indicated that adjunctive tamsulosin therapy plays a critical role in relaxing ureteral smooth muscles, which allows a successful retrograde access for stone extraction and ureteral access sheath placement [24]. Consequently, if a urologist notes the predictive model suggesting stone impaction, alpha-blockers therapy prior to surgery were advocated for these patients.

Present study also has some limitations. Firstly, as a retrospective study, the selection bias cannot be avoided. Secondly, the data from clinical samples were derived from only one center. The validation of the nomogram needs more procedures from other centers. Therefore, in the following research, we will conduct a multicenter external validation with a large number of patients. Thirdly, the slice thickness of NCCT in our hospital is 1.25 mm. The UWT_max_ could be evaluated more accurate by the NCCT with optimal slice thickness.

## Conclusions

This is the 1st attempt at developing a Nomogram to predict using preoperative parameters the possibility of impacted stones. From our study age/past history of stone treatment/degree of hun ( will be ideal to say which grade) and UWT max are the 4 key criteria. This will help urologists to plan and counsel patients for a better ureteroscopic intervention outcome. To our best of our knowledge, this is the first study of a nomogram for predicting impacted ureteral stone prior to treatment. Through this prediction model, urologists can accurately identify impacted ureteral stone and select optimal treatment for patients.

## Data Availability

The datasets used and/or analysed during the current study available from the.

## Abbreviations

URSL: Ureteroscopy lithotripsy
AUROC: Area under the receiver operating characteristic curve
UWT_max_: Maximum ureteral wall thickness
CT: Computed tomography
CRP: C-reactive protein
ESR: Erythrocyte sedimentation rate
NCCT: Non-contrast computed tomography
SWL: Extracorporeal shock wave lithotripsy
PCNL: Percutaneous nephrolithotomy
MPCNL: Mini-percutaneous nephrolithotomy
RPUL: Retroperitoneal ureterolithotomy
